# Cluster Randomized Trial: Sun Protection Intervention ‘Clever in Sun and Shade for Preschools’—Effectiveness and Dissemination

**DOI:** 10.3390/children8080651

**Published:** 2021-07-28

**Authors:** Nadja Seidel, Vera Fieber, Eckhard Wilhem Breitbart, Martin Bornhäuser, Friederike Stölzel

**Affiliations:** 1 Medical Faculty Carl Gustav Carus, The Dresden University of Technology, 01307 Dresden, Germany; ver.fieber@ukdd.de (V.F.); martin.bornhaeuser@ukdd.de (M.B.); friederike.stoelzel@ukdd.de (F.S.); 2 National Center for Tumor Diseases Dresden, University Cancer Center (NCT/UCC), 01307 Dresden, Germany; 3 University Hospital Carl Gustav Carus Dresden, 01307 Dresden, Germany; 4 Association of Dermatological Prevention (ADP) e. V., 20457 Hamburg, Germany; info@professor-breitbart.de

**Keywords:** cancer prevention, skin cancer, UV-radiation, UV-protection, sun protection, preschools, children, outcome evaluation, dissemination, primary prevention

## Abstract

Skin cancer is one of the most common types of cancer and UV radiation is one of the main risk factors. Therefore, sun protection, especially in childhood, is strongly recommended. We examined the effectiveness of the ‘Clever in Sun and Shade for Preschools’ program (CLEVER) in promoting sun protection behavior among preschool staff (trial registration: DRKS00023468) and describe its dissemination. Within a cluster randomized trial with 24 preschools (*n* = 273 staff members) stating a high need for sun protection measures, an educational workshop for preschool staff and a project kit with materials applicable in preschool groups was provided. Staff members of preschools taking part in CLEVER report significantly stronger sun protection behavior to avoid the sun (effect size [ES] 0.70, 95% confidence interval [CI] 0.04 0.71, *p* < 0.05) and less perceived impediments to avoid the sun (ES −0.56, CI −0.82 −0.17, *p* < 0.01) after 12 months as well as higher self-efficacy to avoid the sun (ES 1.09, CI 0.39 1.07, *p* < 0.001) and to use sunscreen (ES 0.71, CI 0.03 0.88, *p* < 0.05) after 1 month. Compared to the control group, there was no significant effect on sunscreen use and further psychosocial outcomes. The effectiveness of CLEVER may be underrated due to a high drop-out rate. Within three years, an enhanced free-of-charge program kit, including a media-based workshop and materials, had reached over 4000 preschools, i.e., 7.1% of all daycare centers in Germany. The results show that CLEVER can strengthen sun protection, offer high-quality information at low cost, and is easily disseminable.

## 1. Introduction

Skin cancer is one of the most common types of cancer and the incidence of melanoma is continuously rising [1]. One of the main risk factors for the development of skin cancer is ultraviolet (UV) radiation [2,3]. Epidemiological data provide evidence of an increased risk of all types of skin cancer being associated with solar UV exposure [3,4]. The contribution of UV exposure during childhood is critical [5]. Due to the special structure of children’s skin, in which skin stem cells are closer to the skin surface up to the age of 12, UV radiation can infiltrate and damage skin stem cells in children more quickly [6,7]. Single events of intensive UV radiation, such as sunburn in childhood, can influence the risk of developing melanoma in adulthood [8,9]. One focus of primary prevention of skin cancer development, therefore, lies in the careful management of UV exposure in children and adolescents [7,10].

In accordance with recommendations of the World Health Organization [2,3], the German guideline on skin cancer prevention recommends a reduction of UV exposure by limiting time outdoors around midday, seeking shade, wearing protective clothes, using sunscreen, and avoiding sunburns as primary prevention measures for children [11]. It lists a number of knowledge-related, behavioral, and environmental measures that need to be considered when promoting sun protection (Table 1).

Several studies have shown that primary prevention significantly reduces the incidence of ‘white skin cancer’ as well as malignant melanoma [12]. However, only few interventions have been designed for the setting of preschools [13]. Most of these studies had limited effects on sun protection behavior [13,14,15], or limitations of the study design [16,17,18]. Examples for successful programs are ‘Sun protection is fun!’ with multiple interventions for preschool staff and parents as well as ‘SunSmart’ with a broader population-based focus [19,20,21]. Overall, the use of age-appropriate interventions using songs and games, for example, improves the knowledge of preschoolers [16,22]. Without the help of adults, however, children of this age are not able to transfer this knowledge into behavior. Including parents and preschool teachers in interventions is especially important for children at an early age, since they control the children’s environment to a large extent, act as behavioral models, and ultimately are supporters for behavior change processes [13,23,24,25]. Current studies show that less than half of the parents of preschool children use sun protection measures correctly and preschool teachers often seem to lack access to adequate information material on sun protection [26,27]. However, measures that are aimed solely at parents and teachers have only limited effects on actual sun protection practices for children [13,28]. Several studies in Germany indicate a reasonable level of knowledge regarding risk factors of skin cancer and sun protection [29,30,31]. Up to 90% of parents are already aware of the increased risk of skin cancer when exposed to the sun [24,30]. This increased knowledge might be a positive effect of sun protection campaigns and awareness programs, but is not automatically transferred into sun protection behavior [32]. Although especially sun exposure avoidance and wearing textiles is recommended, surveys of parents and preschool staff show that primarily sunscreen and hats are used [33,34,35,36], and even parents with a good knowledge of skin cancer risk factors do not adequately protect their children if they have an uncritical attitude towards tanning [37]. Next to focusing on the individuals, the importance of changing relevant settings such as preschools for children has been highlighted [11,38,39]. Sun protection policies often focus on behavioral and environmental measures. Environmental measures are for example technical and organizational interventions, such as the establishment of outdoor areas providing shade in preschools and schools and the adaptation of organizational processes that keep children protected from the midday sun [11,20,40,41]. The UV Index as a risk communication tool, available as digital displays on electronical billboards or accessed via apps and websites, can be useful for improving sun-protective behavior by advising appropriate measures [42,43]. However, interventions aiming solely on the adoption of sun protection policies have limited effects on actual sun protection practices for children [13,28,44], and there is still more research needed on UV Index-related interventions [45]. Overall, interventions designed to last several years and including a large number of settings as well as components such as age-specific curricula and information and training material, have proved to be effective [21,46,47].

When planning a prevention program aimed at children, using a setting-based approach is internationally recommended as well as anchored in German law [39,48,49]. The setting-based approach includes the individual structures of different settings and uses a research-based theoretical framework that involves families, peers, schools, and community partners. Regarding sun protection, a general theoretical foundation, which comprises all determinants for the implementation of appropriate child-centered measures, is not yet available [37,50]. Further recommendations for program-planning comprise long-term and age-specific measures as well as measures that establish policies, institutional, and structural support [39].

In Germany, a ‘Periods-of-Life-Program’ for primary prevention of skin cancer was initiated by the Association of Dermatological Prevention (ADP) e. V. in cooperation with the World Health Organization [10]. It focuses on accompanying young people aged between 0 and 18 years as well as the people responsible for their education. Within a cooperation of German Cancer Aid, ADP e. V., the National Center for Tumor Diseases Dresden and the University of Cologne, the campaign ’Clever in Sun and Shade’ pursues these efforts and combines setting programs for medical practices, preschools, schools, and leisure facilities with media campaigns, involving social and cultural norms as well as legislative and environmental context.

To offer a comprehensive program for preschools that can reduce the risk for skin cancer, the authors developed the ‘Clever in Sun and Shade for Preschools’-program (CLEVER). The program combines theory-based individual as well as environmental interventions and addresses staff members, children and parents. It has been developed to provide materials that effectively promote sun protection and may be easily implemented and disseminated to face the challenges of limited personal and financial resources within both healthcare and educational systems. According to Rabin et al., “dissemination is defined as an active approach of spreading evidence-based interventions to the target audience via determined channels using planned strategies. Implementation is the process of putting to use or integrating evidence-based interventions within a setting” [51] (p. 444). Only few of the numerous cancer prevention interventions that have proven to be effective have been used extensively in practice [51,52]. How to bridge the gap between research and practice and to effectively disseminate and implement prevention programs needs to be explored in more detail [51,53].

This article reports results on the programs’ effectiveness in promoting sun protection among preschool staff. We expected a benefit of CLEVER in the staff’s sun protection behavior and related psychosocial outcomes and in preschool’s written sun protection policy, compared to the sole use of a brochure. Dissemination strategy and implementation of an advanced CLEVER project kit are described and discussed.

## 2. Methods

Adherence to CONSORT guidelines of reporting cluster randomized trials (CRT) is confirmed [54], and the CONSORT-checklist is provided (see Table S1). The study is registered at the International Clinical Trials Registry Platform (DRKS00023468).

### 2.1. Trial Design

This study is based on a CRT with a pre-post-follow up-control group design. Clusters were preschools in Saxony, Germany, with a stratifying variable ‘type of community’. The preschool as institution was the unit of randomization as well as the unit of intervention. Since the intervention focuses on the preschool as institution and the setting changes independently of the participation of every single preschool staff member, the CRT design was selected to evaluate preschool-wide effects of the intervention and maximize the ecological and external validity.

### 2.2. Participants

Preschools as clusters had to meet the inclusion criteria of being located in Saxony, having at least 10 preschool teachers, being interested in participating in CLEVER, not being previously enrolled in a sun protection program, and stating a subjective need for further sun protection measures (i.e., providing sufficiently shaded areas, avoiding the sun, using sunscreen) and information on sun protection. Based on a previous needs assessment with 2145 contacted out of a total of 2237 preschools in Saxony with 653 responses [33], *n* = 52 preschools met the inclusion criteria. Along the stratifying variable ‘type of community’, preschools in each of the four categories (≤5000, 5000–20,000, 20,000–100,000, ≥100,000 inhabitants) were randomly contacted until 6 preschools per category agreed to participate. Recruitment stopped after 32 contacted preschools, when 8 declined and 24 agreed to participate, resulting in a proportional stratified cluster sample with a total of 24 preschools with *n* = 273 staff members (female: 96.7%, age: M = 43.08 years). Participating preschools were randomly assigned to an intervention group or a standard-of-care control group.

### 2.3. Interventions

The intervention was implemented within the clusters in May/June 2016. CLEVER combines theory-based behavioral and setting-oriented measures and incorporates recommendations for primary prevention of skin cancer [10,11,39], addressing all recommendations listed in Table 1 that are appropriate for this setting.

The program aims to support preschool staff in creating an environment that protects children and employees from overexposure to UV radiation. This is approached by promoting sun protection behavior and positive attitudes of staff members towards sun protection as well as stimulating environmental changes and briefing the children. It uses theory-based methods for change, e.g., active learning, participatory problem solving, discussion, and facilitation [38,55,56]. CLEVER consists of an educational team workshop and a project week with ‘Clown Zitzewitz’, the program’s sun protection clown.

The two h team workshop took place within a regular preschool team meeting with all staff members present. Table 2 lists workshop contents and methods for change in detail. A key aspect of the workshop is the development of an individual sun protection strategy for each preschool. This includes the consideration of behavior-related measures for the direct protection of children (e.g., checking whether children are wearing a sun hat/baseball cap), role model-measures for sun protection of preschool staff, environmental-related measures (technical and organizational, e.g., shading by sun shade sails or trees, checking the UV Index, parental support), as well as repeated educational measures for children and staff. Perceived barriers for the implementation of the recommendations are discussed and concise plans of action are drawn up for the preschool team. Group discussions are conducted, focusing on tanning attitudes.

**Figure 1:**
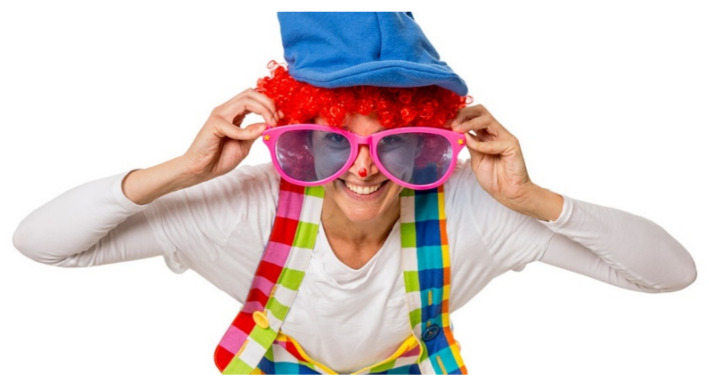
Clown Zitzewitz, the ‘Sun protection clown’ © NCT/UCC 2020.

Subsequently, CLEVER material for a project week is provided. Clown Zitzewitz as a theme runs through the entire CLEVER material for children (Figure 1). The story of Zitzewitz going on vacation and learning the importance of sun protection with the help of his friend Zottelfloh and the children is frequently referred to in CLEVER materials. ‘Theater in health’ can be an effective method in teaching health behavior to children [57], and effectiveness of the theater play ‘Clown Zitzewitz and sun protection’ on children has been investigated [22]. Watching Clown Zitzewitz as a model helps children to witness negative short-term consequences of disregarded sun protection but more importantly to learn how to behave in the sun.

**Figure 2:**
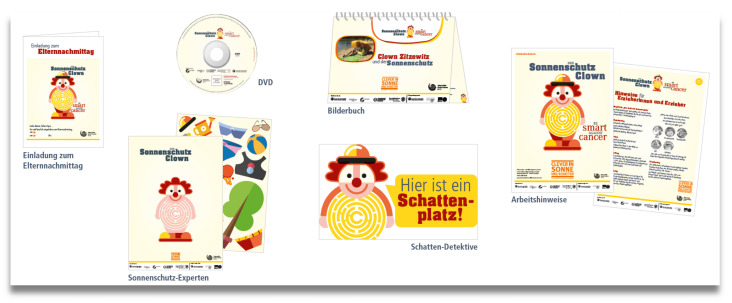
CLEVER material for project week © NCT/UCC 2016.

Within this four-day-project week, children engage themselves with sun protection for at least 1 h per day. The materials include a film- and pantomime-version of the play ’Clown Zitzewitz and sun protection’. Based on the Theory of Social Learning [58,59], Clown Zitzewitz and his friend Zottelfloh act as role models that convey the desired target behavior to the children in a funny and memorable way. The materials also contain the ‘Sun protection-Song’ and a storybook for recapitulating sun protection measures. The riddle on shade and the ‘shade detectives’ exercise help the children to understand the concept of shade and look for spots that are sufficiently shaded in preschools’ outdoor-area. Working on the poster ‘Sun protection experts’, the children decide together what will protect the clown from the sun and attach the cut-out images to the poster. Under guidance of the teachers, children practice the correct application of sunscreen. The parents’ afternoon, which is prepared with invitation cards and holds a performance of the ‘Sun protection song’, marks the end of the project week. Figure 2 gives an overview of CLEVER material. The project week should be established each year on a set date or modified according to the preschools’ needs.

**Figure 3:**
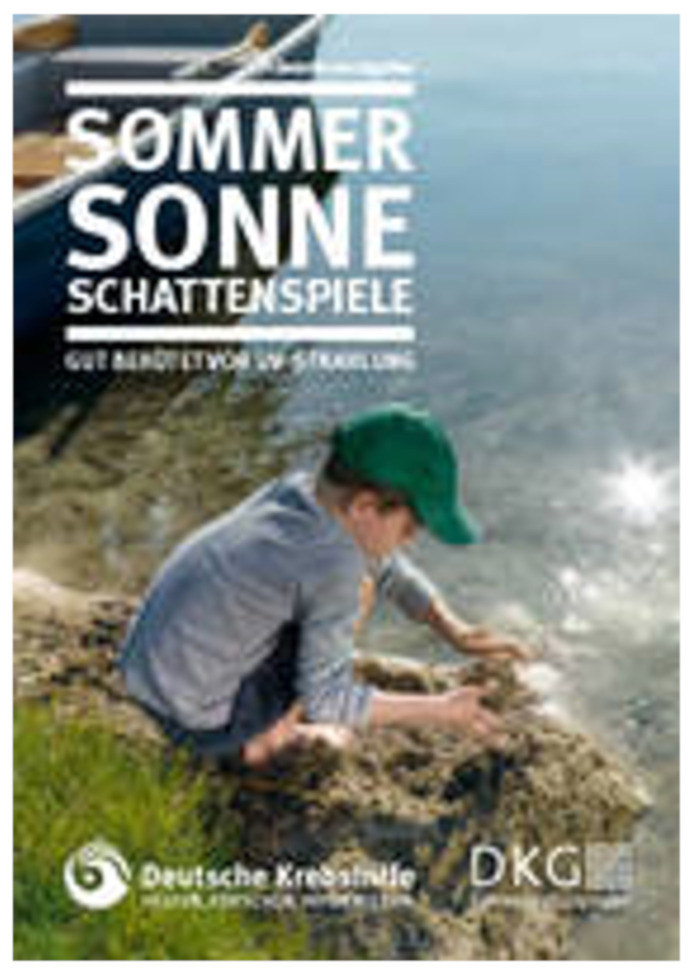
Skin cancer prevention brochure by German Cancer Aid © German Cancer Aid 2016.

Staff members of the control-group preschools received the skin cancer prevention brochure by German Cancer Aid [60] (Figure 3), and were offered CLEVER after the study.

### 2.4. Measures

Baseline, 1-month (medium-term), and 1-year (long-term) assessments were conducted with a paper-and-pencil self-report questionnaire based on previous research (Table 3). The instrument collects data on preschool staff’s UV-protective behavior as primary outcome, relevant psychosocial constructs, and existence of a written sun protection policy as secondary outcomes as well as demographic characteristics.

Based on previous research [20,61], UV-protective measures are divided into sunscreen use and sun avoidance. Sunscreen use addresses the use of sunscreen with a sun protection factor 30+ and regular reapplication. Sun avoidance addresses the use of protective clothes and providing shade. The psychosocial constructs are based on the Theory of Planned Behavior [62], the Social Cognitive Theory [63], and the Health Action Process Approach [64]. Results of factor analyses examining the validity have been reported [61,65]. Scales, reliability, and items of the questionnaire are described in detail in Table 3. To improve reliability, we deleted some of the originally collected items. Only items that are included in the final scales are reported. Most items had 4- to 5-point Likert response scales. Scales were computed as mean scores of the items, except for sun protection policy, which is a sum score.

Behavioral Outcomes were measured based on previous publications [20,61]. Items address the staff’s sunscreen use on their students, students’ use of protective clothing, use of sun shade sails or parasols, and the staff’s own sun protection as part of being a role model on the individual participant’s level. ‘Sunscreen Use Behavior’ (Cronbach’s α = 0.63) and ‘Sun-Avoidance Behavior’ (i.e., protective clothing and shade; α = 0.34) were surveyed on the individual participant’s level.

Psychosocial Outcomes were measured based on previous work [20,37,61,65], and were partly adapted to the Health Action Process Approach [64,66]. ‘Sunscreen Use Self-Efficacy’ (α = 0.62), ‘Sun-Avoidance Self-Efficacy’ (α = 0.58), ‘Health-Related Outcome Expectancies’ (α = 0.74), ‘Appearance-Related Outcome Expectancies’ (α = 0.58), ‘Impediments to Sunscreen Use’ (α = 0.78), and ‘Impediments to Sun-Avoidance’ (α = 0.40) were surveyed on the individual participant’s level. Each self-efficacy scale represented one item for action self-efficacy and one for maintenance self-efficacy. Necessity of sun protection was measured according to Gefeller et al. [31], and addressed the staff’s opinion in five different situations (α = 0.68); all five items were positively correlated.

The Sun Protection Policy was measured based on Crane et al. [14], asking staff whether a written sun protection policy on a preschool level existed (α = 0.76).

**Table 3:** Scales, reliability, and items of the questionnaire.

Scale	Reliability ^1^	Item	Reference
Sunscreen Use ^2^	0.63	I put sunscreen on my students when we go outside.	[61]
		I take sunscreen along when we go on field trips.	[61]
		I use sunscreen with an UV-protection factor of 30+ for my students.	[61]
		I reapply sunscreen on my students every 2 h when we are outside between 11 a.m. and 3 p.m.	[61]
		I put sunscreen on myself when I go outside with my students.	(role model)
Sun-Avoidance ^2^	0.34	My students wear hats or caps when they go outside.	[61]
		My students wear tank tops or halter tops when they go outside. ^5^	[61]
		My students wear long shorts or skirts when they go outside.	[61]
		I take sun shade sails or parasols outside and on field trips so that I can set up shaded areas.	[61]
		When I go outside with my students, I keep to shaded areas myself and wear protective clothing.	(role model)
Psychosocial Outcomes			
Sunscreen Use Self-Efficacy ^3^	0.62	I am confident of being able	
		… to properly apply sunscreen on my students.	[61] (task)
		… to ensure that my students’ parents support me in the provision or use of sunscreen.	(task)
		… to take sunscreen with me on any occasion when my students may be outside.	[61] (maintenance)
		… to get more sunscreen for my students whenever necessary.	[61] (maintenance)
Sun-Avoidance Self-Efficacy ^3^	0.58	I am confident of being able	
		… to ensure that my students’ parents provide them with protective clothing.	(task)
		… to decide if an area is sufficiently shaded to protect my students from the sun.	[61] (task)
		… to check that my students are wearing protective clothes before they go outside.	[61] (maintenance)
		… to provide spare clothes in case the students‘ parents have forgotten suitable clothes.	(maintenance)
Health-Related Outcome Expectancies ^3^	0.74	Avoiding overexposure to the sun protects from premature skin-aging.	[65]
		Avoiding overexposure to the sun decreases the risk for skin cancer.	[65]
Appearance-Related Outcome Expectancies ^3^	0.58	Tanning makes me look better. ^5^	[37]
		Tanned skin is healthy skin. ^5^	[37]
Impediments to Sunscreen Use ^3^	0.78	Putting on sunscreen on my students takes too much time.	[61]
		Putting on sunscreen on my students is always messy.	[61]
Impediments to Sun-Avoidance ^3^	0.40	We do not have enough shade from trees, sun-shade sails, or parasols.	
		Preventing students from taking off protective clothing outdoors is difficult.	
Necessity of Sun protection ^3^	0.68	I think it is important to protect oneself from the sun in the following situations … at the beach. … at noon. … on sunny evenings. … on cloudy summer days. … during outdoor sports activities.	[31]
Sun protection Policy ^4^	0.76	Is it recorded in writing in the facility concept or other documents, that parents are requested … to bring hats or protective clothing? … to provide sunscreen or if the preschool supplies sunscreen? … to provide a written permission for the use of sunscreen?	[14]

^1^ Cronbach’s α; ^2^ Response: 0 = never, 4 = always; ^3^ Response: 0 = strongly disagree, 4 = strongly agree; ^4^ Response: 1 = yes, 0 = no; ^5^ Indicates item was reversed.

*Program exposure* was measured within the 1-month as well as the 1-year assessment. Staff members were asked if they participated in the educational workshop and implemented the project week with the children (for the intervention group) or if they had read the brochure on sun protection (for the control group).

Dissemination strategy and implementation following the study phase is described and evaluated according to the Diffusion of Innovations Theory [67] and the Reach, Effectiveness, Adoption, Implementation, Maintenance (RE-AIM) Framework [68].

### 2.5. Sample Size

Based on a repeated measures ANOVA with two times of measurement, a mean effect size estimate (f (V) = 0.25) of behavior change at the level of preschool teachers with an assumed significance level of α = 0.05 and a power of 0.80, the total sample is 128 preschool teachers (G * Power 3.1) [69,70]. Intraclass correlation coefficients (ICCs) in CRTs in preschools and schools ranged from 0.05 to 0.30 [61,71,72,73]. We anticipated a design effect due to an ICC of 0.15 and a mean cluster size of 10 [74], as well as a drop-out of 25%; thus 240 preschool teachers, i.e., 24 preschools, were required.

Post hoc sensitivity analysis revealed that, considering a design effect due to an ICC of 0.008 for our changes in the primary outcome, a mean cluster size of 6.33 (SD 4.70) and the correction of the design effect for the coefficient of variation of cluster sizes CV = 0.74 [75] within an ANCOVA with two covariates, a medium to large effect size of f (V) = 0.37 could be detected with a power of 0.80 and a significance level of α = 0.05 [69].

### 2.6. Randomization

Preschools meeting all inclusion criteria were randomized (1:1) with stratified random sampling. The stratifying variable was ‘type of community’ (≤5000, 5000–20,000, 20,000–100,000, ≥100,000 inhabitants) of the preschool. All institutions responsible for the preschools gave their consent before randomization. A blind randomization was carried out by running a randomization script over the list of encrypted preschool codes. Allocation sequence, preschool enrollment, and assignment to intervention were performed by the study investigators. Informed written consent to participation was required from the preschool teachers. All educational staff members of participating preschools with a written consent form were included. Blinding was not possible after assignment to either control or intervention group.

### 2.7. Statistical Methods

To evaluate changes after intervention regarding sun protection behavior, impediments and sun protection policy (change baseline–1-year assessment) as well as necessity of sun protection, self-efficacy and outcome expectancies (change baseline–1-month assessment), we conducted linear mixed-effects models (LMM) on change scores with treatment group as fixed effect, preschool as random effect, as well as corresponding baseline scores and age as covariates. Outliers within the change scores were replaced by mean-score plus two times the standard deviation [76]. One-way analyses of variance (ANOVA) were used to compare intervention and control group at baseline. We computed ICC for the primary outcomes out of the ANOVA estimator [77] and provide 95% confidence intervals (CI) for effects [78]. For further exploratory analyses, we report descriptive statistics of baseline item responses and LMM-results on single items. Parallel multiple mediation analyses were performed, using the PROCESS macro Version 3.5, to predict changes in sun protection behavior with intervention group as independent variable and changes in self-efficacy, outcome expectancies, impediments, necessity of sun protection and sun protection policy as mediators regarding sun avoidance and sunscreen use, respectively [79,80]. Two-tailed tests were used and all statistics were performed on an intention-to-treat basis using SPSS, Version 27.0 [81].

## 3. Results

### 3.1. Participant Flow

**Figure 4:**
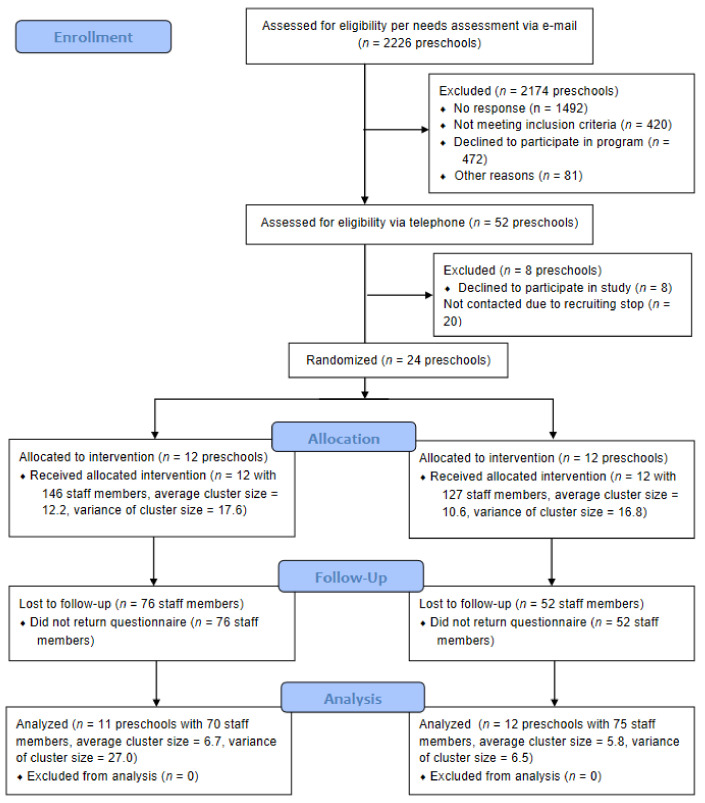
Flowchart for enrollment, baseline measurement, and follow-up.

The flowchart (Figure 4) describes the numbers of clusters as well as staff members that were randomly assigned, received intended treatment, and were analyzed for the primary outcome. 54% of staff members were lost to follow-up (1-month and 1-year assessment). Significant differences in the baseline scores of Sun-Avoidance Self-Efficacy (mean difference: 0.15, CI 0.00 0.29, *p* < 0.05) and Health-Related Outcome Expectancies (0.29, CI 0.04 0.54, *p* < 0.05) were found with higher scores for the drop-outs.

### 3.2. Recruitment

Recruitment took place from November 2015 to March 2016. We surveyed preschool staff in April/May 2016 (baseline), one month after the intervention in June/July 2016 (1-month assessment), and one year after the intervention in June 2017 (1-year assessment).

### 3.3. Baseline Data

Table 4 contains baseline information on demographic characteristics and outcome scores for intervention and control group. No significant differences were found between the two treatment groups.

### 3.4. Outcomes

Reliability for the subscales ranges from good for ‘Impediments for Sunscreen Use’ (α = 0.78) and ‘Sun Protection Policy’ (α = 0.76) to low, especially for ‘Sun-Avoidance Behavior’ (α = 0.34) and ‘Impediments for Sun-Avoidance’ (α = 0.40), possibly reflecting the diversity of the construct ‘Sun-Avoidance’. For changes in ‘Sun-Avoidance Behavior’, ICC = 0.001 (for baseline score 0.09) and for changes in ‘Sunscreen Use Behavior’, ICC = 0.008 (for baseline score 0.14). No adverse events or harms were reported.

#### 3.4.1. Program Exposure

At the 1-month assessment, 73% of staff members in the intervention group reported their workshop attendance and 72% reported to have implemented the project week at least partly. Of the control group, 59% reported having read the brochure at least partly. At the 1-year assessment, 62.8% of staff members reported to have implemented the project week at least partly anew in the second year.

#### 3.4.2. Intervention Effects

Table 5 presents changes in behavioral and psychosocial outcomes and sun protection policy by the treatment group. After adjustment for baseline score and age and controlling for random effects of preschool, preschool staff members showed a significant increase in their behavior to protect children from the sun by avoiding the sun, i.e., providing shade and using protective clothes (*p* < 0.05). There was no change in the use of sunscreen. Regarding psychosocial outcomes, self-efficacy regarding sun-avoidance (*p* < 0.001) as well as ‘regarding the use of sunscreen’ (*p* < 0.01) increased significantly. Concerning UV-protective behavior, impediments to sun avoidance (*p* < 0.01) but not impediments to sunscreen use (*p* = 0.88) decreased in the intervention group. There was no significant treatment effect regarding outcome expectancies (*p* = 0.48, *p* = 0.86), necessity for sun protection (*p* = 0.21) and sun protection policy (*p* = 0.58).

Exploratory ancillary analyses of changes in single items of sun protection behavior show no significant group differences: put sunscreen on when outside (*p* = 0.98), take sunscreen on field trips (*p* = 0.85), use of sun protection factor 30+ (*p* = 0.60), reapply sunscreen every 2 h (*p* = 0.43), put sunscreen on myself when getting outside with students (*p* = 0.50), students wear hats or caps (*p* = 0.19), students wear tank tops (*p* = 0.38), students wear long shorts/skirts (*p* = 0.60), staff set up shaded areas (*p* = 0.35), keep to shaded areas myself, and use protective clothing when getting outside with students (*p* = 0.07).

To describe the sun protection behavior, attitudes, and further variables, baseline ratings of the whole sample are reported (Table 6).

**Figure 5:**
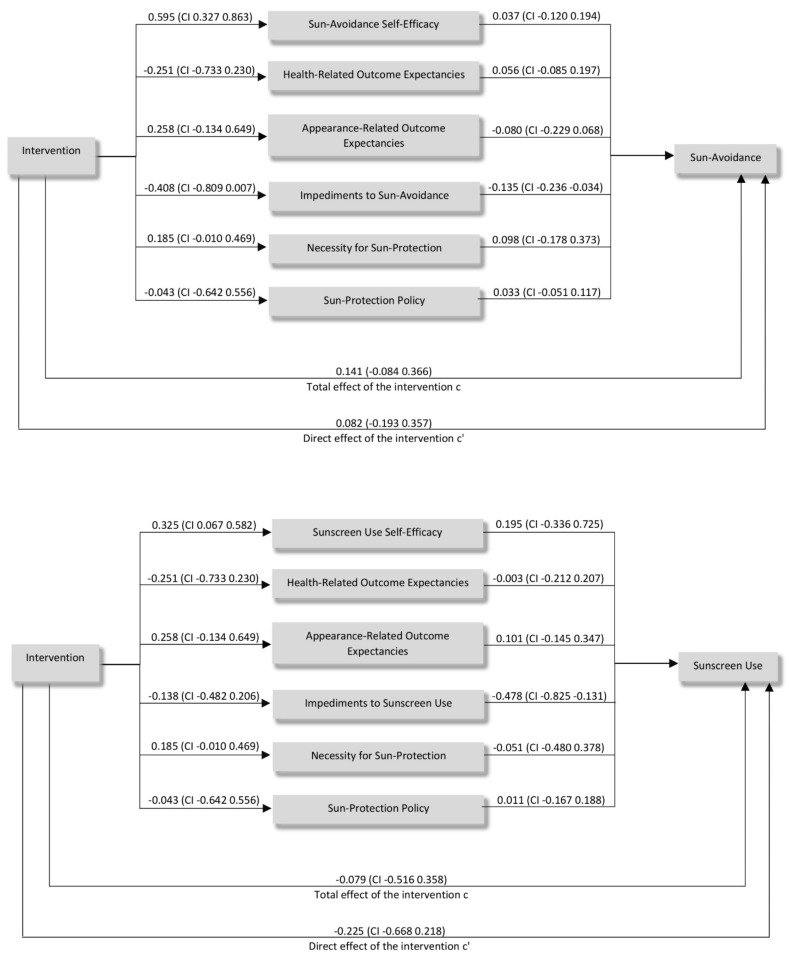
Mediation analyses pathways for changes in Sun-Avoidance Behavior and Sunscreen Use Behavior.

Figure 5 shows the pathways of the mediation analyses. The relationship between CLEVER and change in Sun-Avoidance Behavior is mediated by a change in Impediments to Sun-Avoidance (indirect effect = 0.055, CI 0.001, 0.143), but not by changes in Sun-Avoidance Self-Efficacy (0.022, CI −0.072, 0.118), Health-Related Outcome Expectancies (−0.014, CI −0.094, 0.014), Appearance-Related Outcome Expectancies (−0.021, CI −0.072, 0.026), Necessity for Sun protection (−0.018, CI −0.025, 0.099) and Sun protection policy (−0.001, CI −0.035, 0.035). Participating in the CLEVER intervention significantly reduced impediments towards sun-avoidance behavior and reduced impediments were significantly associated with better sun-avoidance behavior. Furthermore, we found the relationship between CLEVER and a change of Sunscreen Use Behavior is not to be mediated by changes in Sunscreen Use Self-Efficacy (indirect effect = 0.063, CI −0.083, 0.220), Health-Related Outcome Expectancies (0.001, CI −0.075, 0.071), Appearance-Related Outcome Expectancies (0.026, CI −0.046, 0.118), Impediments to Sunscreen Use (0.066, CI −0.115, 0.227), Necessity for Sun protection (−0.010, CI −0.113, 0.063), and Sun protection Policy (−0.001, CI −0.063, 0.047).

#### 3.4.3. Dissemination and Implementation of CLEVER

**Figure 6:**
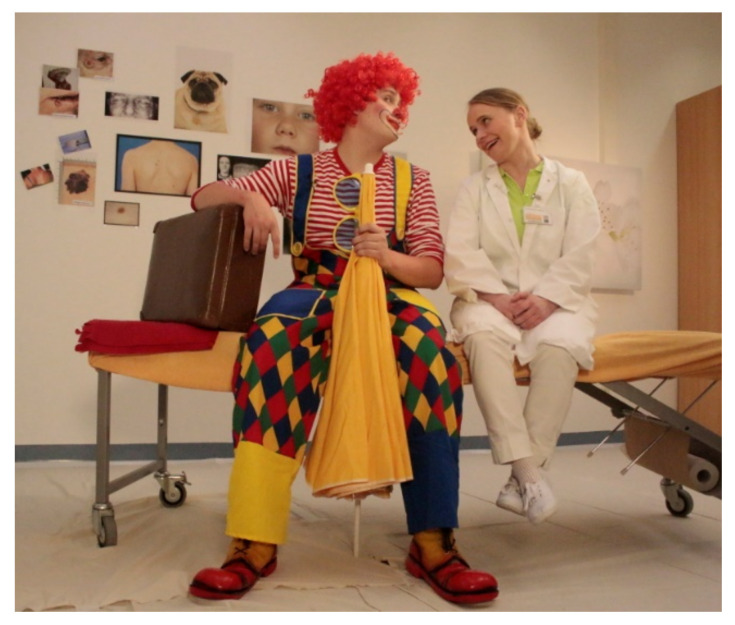
Clown Zitzewitz and dermatologist in the film of the media-based workshop © NCT/UCC 2018.

**Figure 7:**
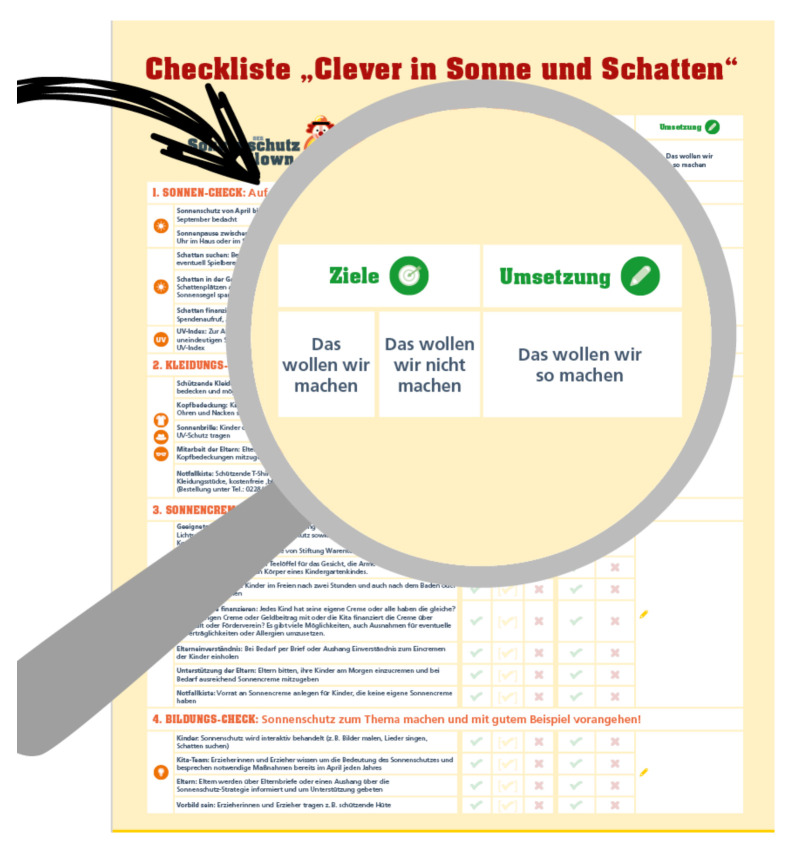
CLEVER-checklist fosters goal-setting and detailed planning for the institutions’ individual sun protection strategy © NCT/UCC 2018.

After study completion, the intervention was adjusted to reduce personnel expenses for the educational workshop and therefore reduce costs and facilitate the program implementation. Based on the experiences of the educational workshop, an interactive media-based workshop as well as a checklist for the sun protection strategy was developed. The media-based workshop uses the filmed story of Clown Zitzewitz seeking the advice of a dermatologist on his latest sunburn (Figure 6). In several sequences, Zitzewitz and the expert cover several “prototypical” attitudes in favor of or against sun protection. The film is supposed to promote team discussion on the subject of sun protection. The checklist supports the preschool team to develop their own sun protection strategy by analyzing the status quo, setting goals, and fostering detailed planning (Figure 7; for full checklist see Figure S1).

**Figure 8:**
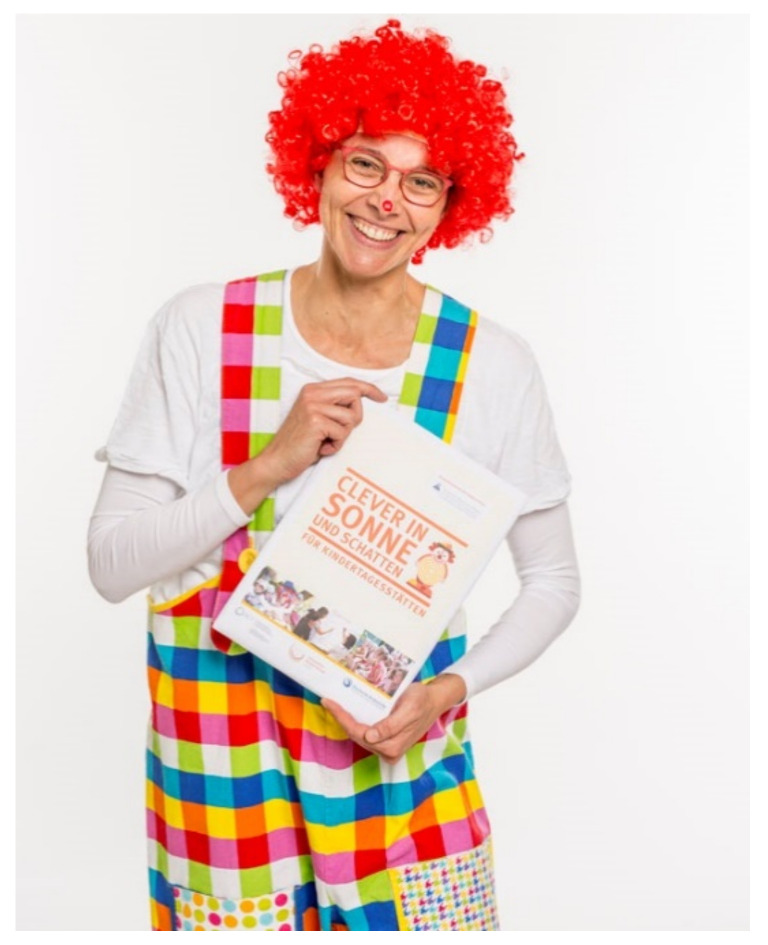
CLEVER project kit for mailing © NCT/UCC 2018.

**Figure 9:**
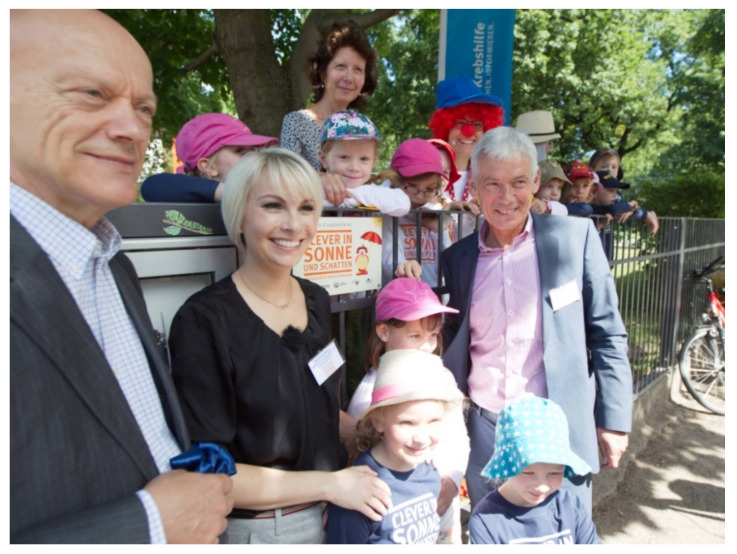
The first of more than 250 CLEVER Awards © Deutsche Krebshilfe 2017.

**Figure 10:**
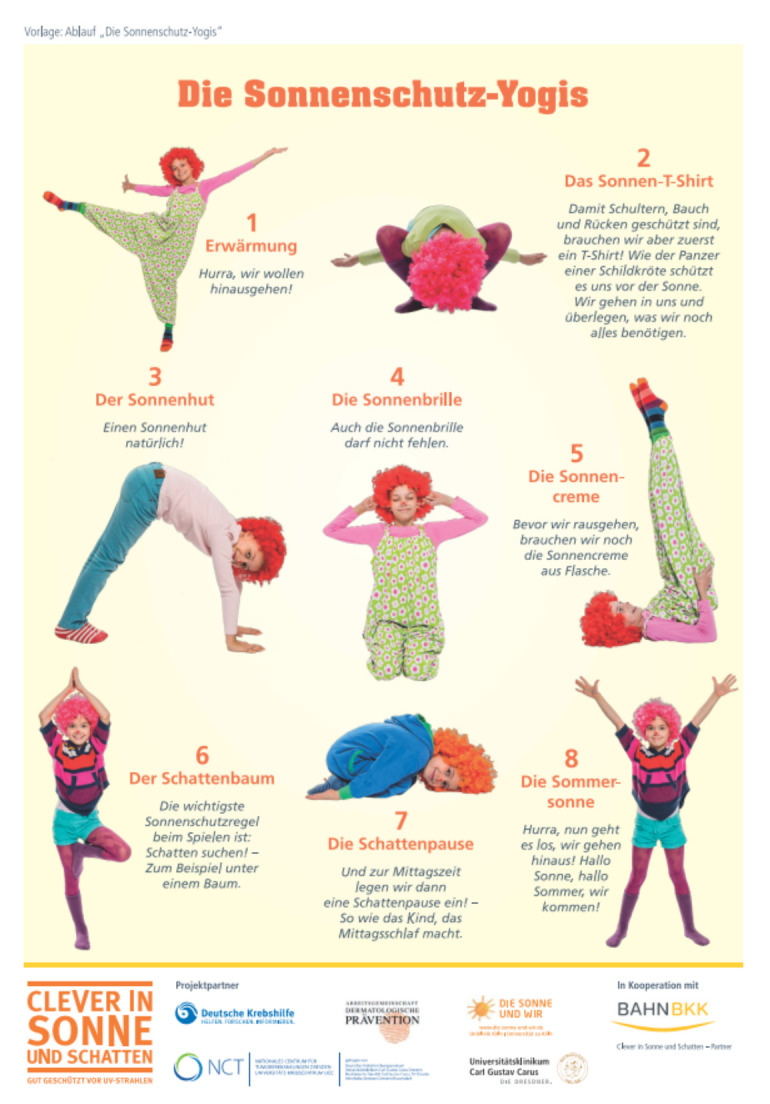
Additional low-threshold yoga-material © NCT/UCC 2021.

Since 2018, CLEVER has been available as a project kit guiding preschools to implement a comprehensive sun protection intervention (Figure 8). It consists of a media-based interactive educational workshop for preschool-staff (DVD and checklist) as well as material applicable in preschool groups and is mailed to interested institutions free-of-charge. A preschool that has conducted and documented the CLEVER workshop for its staff as well as the project week with the children, can receive the ‘Clever in Sun and Shade’-Award. This contributes to the institutions’ self-commitment to maintain sun protection measures and illustrates the importance of sun protection and skin cancer prevention to parents and the community (Figure 9). In addition to the visible award, a lottery of funds for trees and sun shade sails for awarded preschools is used as an incentive. Multipliers can also be awarded as ‘Clever in Sun and Shade’-partners. New material for the project week is constantly developed and offered to preschools to (1) address further sub-target groups or related target groups (e.g., finger play and picture book for children < 3 years, experiments for kids in pre-primary education or elementary school grade 1 & 2), (2) set incentives to repeat the project week each year with novel material, and (3) provide further low-threshold material. For these additional and low-threshold material, ideas of preschools as well as recent trends in education are considered. One example is recent material for yoga with Clown Zitzewitz, which might be especially appealing to a subgroup of preschool teachers (Figure 10). Yoga materials can be implemented in pre-primary education within preschools beyond a project week but also work well for media campaigns.

For dissemination, communication objectives according to the diffusion of innovations model were created to make the case that CLEVER: (1) conveys current recommendations on sun protection for the living environment of children (relative advantage); (2) is theoretically sound and scientifically supported, and free of advertising (compatibility); (3) enables flexible, independent implementation of various project modules (complexity); (4) is free-of-charge (trialability); (5) provides guidance on sustainable implementation in everyday life and makes long-term commitment visible with an award (observability) [82].

**Figure 11:**
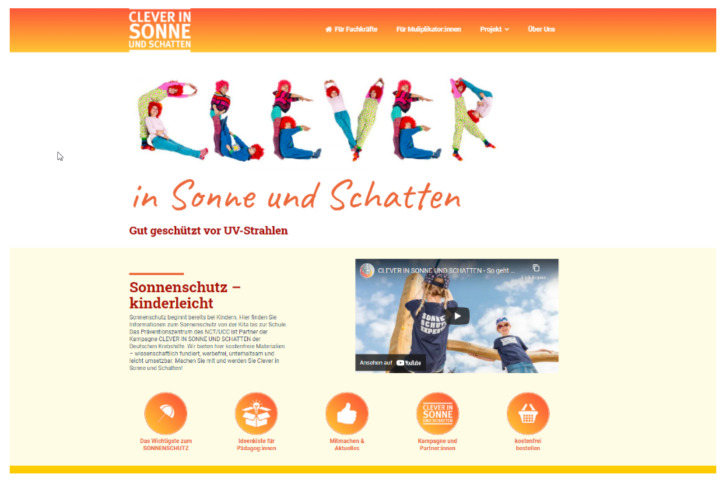
Program website www.cleverinsonne.de © NCT/UCC 2021 (accessed on 27 July 2021).

Dissemination methods used in CLEVER are constantly adapted and elaborated. They aim at district officials; education and health department staff as multipliers; as well as preschool teachers, managers, and parents as persons responsible for youth education. These methods comprise emailing, telephone contact, presentations on public events, displays at various conferences, websites (Figure 11), advertising in publications relevant to target groups and word-of-mouth referrals. Cooperations are established with statutory accident insurance companies, who are responsible for preschool settings in Germany, and health insurance companies, whose task amongst others comprises the support of primary prevention measures to motivate and enable individuals to keep themselves healthy. The CLEVER team also targeted its dissemination activities to social media change agents such as Susanne Klehn, an anchorwoman and skin cancer patient herself, and the ambassador for skin cancer prevention for German Cancer Aid. Opinion leaders were also targeted, including researchers and state, federal, or private organizations responsible for children’s health by giving presentations at state, national, and international meetings and making direct contact with key change agents.

Costs for printing and mailing per project kit are about 5 €, which are covered by German Cancer Aid. Over three years, more than 4000 preschools out of 55,900 daycare centers have ordered the program. Thus, 7.1% of all German daycare centers with 44,000 preschool teachers and 260,000 children have potentially been reached. Cooperation with statutory accident insurance companies led to a locally higher reach and thus a proportion of participating preschools. The absolute number and proportion of preschools that implement the program is not known. Feedback is gained only from preschools that register for the award, which were 271 preschool adopting the program. Out of these, 258 have successfully been awarded and 13 were declined due to insufficient program fidelity. For these 258 awarded preschools, program fidelity was around 90%. Maintenance has not been measured, but awarded preschools make a commitment to implement CLEVER every year.

## 4. Discussion

Unlike congenital risk factors such as skin type, personal UV exposure can be influenced to a significant extent by behavior and external circumstances. Since “childhood is believed to be a susceptible window for long-term harmful effects of UV, […] effective UV radiation protection from childhood is necessary to control both immediate and long-term harmful effects on children’s skin” [7] (p. 349). Lessons learned from previous studies and programs are that sun protection education has to be accompanied by behavioral as well as environmental measures and vice-versa [13,21,38,39,47]. Up to date, only few studies have investigated the effects of sun protection interventions in a preschool setting using randomized controlled trials or CRTs [83,84]. A minority of these evidence-based programs are still continued, such as the best-known and well-studied SunSmart Schools and Early Childhood Membership Programs in Australia, as well as the US program Ray and the Sunbeatables™, which is based on the ‘Sun Protection is Fun’ intervention [21,85,86].

The CLEVER study is the first CRT in Germany to investigate a sun protection preschool setting intervention aiming at individual behavior and environmental changes in a sample with a stated high need for sun protection measures. Furthermore, it is the first nationwide German sun protection program being embedded in a larger focus addressing young people and has explicitly been developed to be easily disseminated. Due to a high drop-out rate, only medium to large effects could be detected. This may underrate the effectiveness of the CLEVER program. Furthermore, the external validity may be limited by the drop-out. Childcare institutions often undergo a high staff turnover [20,87]. The impact of CLEVER may be restricted to staff members being present at the implementation of the intervention.

### 4.1. Effectiveness

At the 12-month assessment, staff members in intervention preschools were more likely to protect their students by avoiding the sun and stated lower impediments to sun-avoidance than staff members in preschools that received an information brochure. Furthermore, significant intervention effects on self-efficacy to avoid the sun and to use sunscreen were found at the 1-month assessment. No significant intervention effects were detected for sunscreen use behavior and its impediments, health- and appearance-related outcome expectancies, the necessity for sun protection, and the preschool’s sun protection policy. The positive treatment effects, particularly for avoiding the sun, may be a result of the consistent message that states that these measures are recommended before sunscreen use. These findings are in line with recommendations of skin cancer prevention [11] and are therefore greatly appreciated.

Despite CLEVER’s overall significant effect on sun-avoiding behavior, there were neither significant intervention effects on the single measures of wearing a hat or protective clothing by the students, of setting up shaded areas nor of acting as a role model by avoiding the sun oneself. This suggests a long-term benefit of CLEVER in the general concept of avoiding the sun, possibly adding up through smaller changes. Even if James et al. confirmed the validity of a similar scale [61], the scale seems to be heterogeneous since internal consistency is quite low. Regarding the effect on the use of sunscreen, neither a general effect of the intervention nor a benefit on the single measures of the use of sunscreen, the sun protection factor, taking sunscreen on field trips, and acting as a role model by using sunscreen oneself could be found. Interventions similar to CLEVER reported variable outcomes. The program ‘Sun Protection is Fun’ improved use of sun-protective clothing, shade provision, as well as sunscreen use [20]. For the US program, ‘Block the sun, not the fun’, only behavioral effects for sunscreen use were seen [14]. Members of the Australian SunSmart program reported more sun protection practices and over the decades, most sun protection practices such as the use of sunscreen, hats, and sun-protective clothing were used by an increased proportion of all nationwide early-childhood services [88].

According to the Health Action Process Approach and several studies, actual health behavior is built up by pre-intentional motivational and post-intentional volitional processes [64]. Within pre-intentional processes, the belief in one’s capability of using sunscreen or avoiding the sun (self-efficacy) is as important as the belief of positive health- and appearance-related consequences for building an intention for sun protection practices, even for the protection of children [37,64,65]. Within our intervention, we were able to promote the preschool staff’s perceived self-efficacy regarding sunscreen use and sun-avoiding behavior, but could not promote positive outcome expectancies. Whereas the belief of sun protection behavior on positive health consequences has been already high at baseline, the median moderate belief of positive appearance-related consequences may counteract the intervention’s benefits in some individuals or even preschools and thus impede appropriate sun protection behavior. In a study by Gritz et al. [20], tanning attitudes were only affected after 24 months rather than after 12 months, pointing out that it possibly requires even more time to change these attitudes. Once an intention is built, the “good intention” has to be transformed into a detailed plan on how to perform it [64]. Within our intervention, this ‘action planning’ of sun protection measures is anchored in the development of a detailed sun protection policy. By this, even if a staff member has “no good intention” on the individual level, it may be obliged to carry out the desired behavior within the preschool. According to the Health Action Process Approach, the anticipation of barriers is also an important component of planning [64]. The ‘coping planning’ is the imagination of possible barriers which generates strategies to overcome them [64]. Within our team workshop, barriers to adequate sun protection were pointed out and the team discussed ways to overcome these barriers. The individual’s maintenance self-efficacy, which represents the beliefs about one’s capability to deal with barriers that arise during the maintenance of behavior, is also important [64].

The presence of a written sun protection policy and necessity of sun protection were not changed by CLEVER. This may be due to a mismatch in the items of these scales and the intervention contents. The sun protection policy within CLEVER focuses on more measures than written instructions for the work with parents, i.e., behavioral, technical, and organizational measures. The dependence of preschool staff on parents to provide hats and clothes to protect children as well as the need for more parental contribution and sponsors to supply and finance sunscreen has been highlighted earlier [20,33]. In a German study, a majority of preschools (86%) had sun protection rules, while only a minority (18%) provided a written policy [87]. Guidelines concerning seeking shade and avoiding peak sun intensity hours were stated less often than wearing hats and applying sunscreen, while long-sleeved clothing and sunglasses were rarely or never mentioned [87]. However, recent studies in preschool and primary school settings in Australia show the potential impact of sun protection strategies on sun protection practices [85,88,89]. In contrast to Germany, about 86% of all Australian early childhood services provide a recent sun protection policy [88], and the development of a sun protection strategy has been shown to be associated with better sun protection behavior of children and staff [89]. Constant change in staffing and leadership, but also in children and parents, has been described as a barrier to implementation [90]. Staff turnover in childcare centers is commonly high and therefore the long-term impact of skin cancer prevention programs is still unclear [87]. To enhance successful implementation, strategies that reinforce key behavioral messages and that are accessible for new staff are required [90]. Besides repeated interventions, the development of a written sun protection policy is recommended, since it may secure the implementation with a high standard [87]. Further research on CLEVER may benefit from the adaption of the questionnaire according to these investigations, i.e., the comprehensive measurement of child-related sun protection practice criteria (hat-wearing practices, sunscreen practices, and protective clothing practices) as well as organizational-level sun protection practices (enforcement of policy, role modelling, education, shade provision, policy review and update, information for caregivers) [88].

The necessity of sun protection at the beach and on outdoor sports activities were part of the scale but not of the curriculum and could therefore not be enhanced. The focus of the CLEVER-curriculum on everyday settings is in line with the findings of a survey among German parents. There is an apparently a lower subjective need to protect children in everyday outdoor situations in contrast to beach settings [91]. It is worth pointing out the necessity of sun protection on cloudy summer days. In accordance with a study on parents [31], staff members overrated the protective effect of clouds. UV radiation may even pass through a thin layer of cloud cover [11], and it is therefore a common mistake to neglect sun protection on cloudy days. Overall, more research is needed for the validation of knowledge or subjective necessity of sun protection and sun protection policy measurements. Both aspects may, besides epidemiological data on skin cancer and sun protection behavior, contribute to describe possible effects and reach of sun protection programs and campaigns within a larger scope [10,31,44,88].

Possible intervention mechanisms can be evaluated using mediation analysis. We assessed self-efficacy, outcome expectancies, impediments, necessity for sun protection, and sun protection policy as mediators for the intervention. The results suggest that less perceived impediments may have moderated the CLEVER effect on sun-avoidance behavior. However, this finding should be interpreted with caution, since observations are dependent on the CRT design; multiple mediators that affect one another may act as confounders; and reverse causation could be existent because the mediators were partly measured at the same time as the outcome [80,92]. In line with this, Hunkin and Morris point out that there might be substantial barriers in implementing specific sun protection practices by daycare centers, limiting the effectiveness of the interventions, and that there is a need for future research on these impediments [88].

What should be considered to further enhance sun protection programs? Baseline measurement displays a need to support preschool staff to promote protective shirts and shorts/skirts, to put on sunscreen, and to act as a role model regarding sun-avoidance and sunscreen use. Furthermore, staff especially needs to be encouraged to reapply sunscreen at appropriate intervals and to take sunscreen on field trips. Measures that are reported to be well implemented are ‘students wearing caps or hats’, ‘staff takes sun shade sails or parasols outside and on field trips to set up shaded areas’, and ‘the use of sunscreen, even with a sun protection factor of 30+’. We have not collected data of sufficiently shaded areas. Several surveys show similar results, pointing out that primarily sunscreen and hats are used [33,34,35,93]. Moreover, staff turnover, sun protection policy development, impediments, tanning attitudes, and the overestimation of the UV-protective effect of clouds should be paid more attention to in future programs.

### 4.2. Dissemination

A significant number of children can be reached via programs in preschools. In Germany, more than 92% of children aged 3–6 are cared for in 55,900 preschools, i.e., more than 2 million children [94]. With our free-of-charge program kit, we reached 7.1% of all German day-care centers within 3 years, representing potentially 44,000 preschool teachers and 260,000 children. This was only possible by adapting the original educational workshop to a media-based interactive workshop that could be implemented independently by the preschool. Furthermore, funding for the project kits is necessary to offer it free-of-charge and to reduce barriers to order it. In order to spread information on CLEVER, being free-of-charge and free of advertising was an important characteristic for our main multipliers, the education and health department staff. For funding, on the other hand, the most important characteristics were being in line with national recommendations of sun protection and considering the setting approach that is anchored in national law.

The effectiveness of the current mail-only intervention with its media-based workshop, however, has to be further considered. An evaluation is currently underway. Other mail-only dissemination strategies with policy guidelines showed strong effects for the adoption of a sun protection policy but were ineffective in promoting sun protection practices [28,44]. Other criteria of the RE-AIM Framework such as implementation, adoption, and maintenance will have to be further evaluated in an implementation study [68].

Currently, there exist only a few evidence-based cancer prevention programs that have been extensively utilized in real-world preschool settings. There is the outstanding ‘SunSmart’ program in Australia with a broad population-based focus. Its multi-setting approach focuses on behavioral, environmental, and legislative changes and, together with media campaigns over many decades, appears to have resulted in the decrease of melanoma incidence [21]. One recent statewide effort for sun protection in the United States in preschool, kindergarten, and first-grade students is the ‘Ray and the Sunbeatables’™ program by the MD Anderson Cancer Center [21,95]. Within its implementation study, “observed curriculum adaptations and varied preschool contexts, highlight the need to consider fidelity of implementation of sun protection concepts and behaviors, and not exclusively fidelity of implementation of program components” [86]. Lack of time and change of staff turned out to be barriers to sustained implementation [90]. Redefining these barriers as opportunities, CLEVER offers additional material such as ‘sun protection yoga’ that regards fidelity of implementation of sun protection concepts rather than fidelity of the whole program. Therefore, new staff may be attracted by the low-threshold materials. Furthermore, a program during preschool teacher training is currently set up to reach young professionals. It may gradually support a change of culture that is required for educational institutions to accept sun protection as a duty of care and to implement not only regulatory measures and healthy policies but internalizing that leading by example may help protect students and staff from UV exposure during care time [38,96].

### 4.3. Limitations

The findings of the study may be limited to preschools that stated a high subjective need. However, baseline assessment of sun protection measures were similar to other surveys [35]. The study holds a high drop-out rate of 54% of staff members from baseline to 1-year follow-up. This might lead to an underestimation of the effectiveness of CLEVER, resulting in a sensitivity for medium to large effect sizes only. Besides reducing the power of the study, dropping out may threaten validity. Regarding internal validity, there is no indication that the frequency or the causes of dropping out differ between the intervention groups and therefore no indication that the results are biased by a differential drop-out [97]. Regarding external validity, drop-out in longitudinal studies threatens validity because participants that complete the trial might differ from participants that drop out during the trial. Significant differences in the baseline scores of Sun-Avoidance Self-Efficacy and Health-Related Outcome Expectancies were found, with slightly higher scores for staff members that dropped out early. These findings might indicate that a higher percentage of staff members with slightly more favorable attitudes towards UV protection was among the study participants that dropped out early. One reason for the high drop-out rate might be the high staff turnover, which is quite common in childcare institutions [87], and which may affect external validity. CLEVER results may be restricted to staff members that are present at the implementation of the intervention. To handle high staff turnover within a study, a cross-sectional approach might be more appropriate [20]. Another reason for the high drop-out may be the distribution of questionnaires via preschool management. Since the participation of staff members was anonymous, we provided plain envelopes and only few directors recorded who returned the questionnaire. Conducted reminders had low success. The reliability of some subscales, especially for behavior and impediments for avoiding the sun is quite low. Cronbach’s α of ‘Sun-Avoidance Behavior’ would have improved with the deletion of the item ‘students wear tank tops/halter tops’, but we decided to insert the item since wearing protective clothes is a crucial sun protection measure. Even if the construct is considered heterogeneous, the scale might be rethought. Social desirability bias as well as recall bias limit behavioral self-report methods. However, self-report by caregivers on child-centered sun-exposure-related variables is considered valid and reliable [98,99]. We did not correct significance levels for multiple tests, since post hoc sensitivity analysis showed that the study was powered only for medium to large effect sizes. Contrary to our expectations, sun protection measures differed not between the type of community in Saxony; therefore, a CRT sample without stratification may be more appropriate [33]. Data has not been collected from students in this study, thus no statement can be made about the children’s sun protection behavior and how it is related to the sun protection behavior of staff members. The CLEVER trial lasted 15 months. Gritz et al.’s findings indicate that changing attitudes towards sun protection may require more time [20]. Future studies should be designed with a broader time-frame.

## 5. Conclusions

Only a limited number of sun protection programs are evidence-based and have been utilized in real-world preschool settings. Evaluation results of our CRT show that CLEVER is a very promising program to sustainably promote sun protection in preschools. It is superior to the distribution of an information brochure concerning crucial outcomes, with medium to large effects on the actual behavior of staff members and important predeterminants of behavior change. The high drop-out rate limits the power of the study and may reduce generalizability. A further program development, the mail-only intervention with its media-based workshop, increases flexibility of the implementation and is already utilized. Over three years, the enhanced free-of-charge program kit has reached 7.1% of all daycare centers in Germany. The results show that CLEVER offers high-quality information at low cost and is easily disseminable. CLEVER engages in finding solutions for implementation barriers such as lack of time and change of staff. Additional low-threshold material to attract participants and to reinforce key behavioral messages is provided. The effectiveness of the current mail-only intervention, its implementation, adoption, and maintenance will be further evaluated. 

## Figures and Tables

**Table 1:** Recommendations for measures promoting primary skin cancer prevention, according to the German guideline on skin cancer prevention [11].

Type of Measure	Recommendation
Knowledge-related	Educational measures on UV radiation and protective measures in kindergarten/preschools and schools can improve knowledge about sun protection.
	UV-risk communication should address aspects relevant to everyday life, the subjective perception of gain by UV exposure and the ideal of beauty of tanned skin. An important starting point for communication should be social ideals and behavioral routines with regard to tanned skin and sunbathing.
	The range of media information on skin cancer prevention should be expanded qualitatively and quantitatively, since the media are the most important source of information for adults.
	Digital media literacy as part of the population’s health literacy should be promoted in order to be able to find, understand, and assess the quality of information on skin cancer and skin cancer prevention in a more targeted manner.
	Parents with children in kindergarten/preschool as well as educators, teachers, and daycare center/preschool managers should be informed about UV radiation as a risk factor for skin cancer and about the inadequate protective function of clouds against UV radiation.
Behavioral	Interventions aimed at influencing behavior over the long term should consist of several components, be intensive, and designed to be repeated.
	Behavioral change interventions should be based on behavioral theories and should take available evidence into account.
	Measures to convey primary prevention of skin cancer should be multimedia-based as well as interactive and integrate several communication channels.
	Information can be provided through parents, teachers, educators, peers, and other multipliers.
	Skin cancer prevention interventions that also address external appearance are a promising strategy for changing sun protection behavior.
	Measures for primary prevention of skin cancer should be designed with a focus on the target group and take the target group’s needs into account.
	In order to reach people where they shape their everyday lives, measures for primary prevention of skin cancer should be setting-related.
	Sun protection interventions for children and adolescents should be conducted in preschools and schools.
	The UV Index should be more intensively communicated and used in sun protection recommendations and programs.
Environmental	A sufficient amount of shaded areas should be provided in kindergartens, preschools, and schools.
	Technical and organizational measures to avoid excessive UV exposure, especially during the midday hours (e.g., provision of shaded areas, consideration of sun protection when scheduling sporting events for example) should be an essential part of primary prevention.
	Evaluation: Primary skin cancer prevention interventions should be evaluated formatively and summatively. The evaluation parameters used should be derived from a theoretically proven model.

**Table 2:** Scope, content, and methods for change of the CLEVER team workshop.

Scope	Content	Methods for Change
Promoting staff’s knowledge, attitudes and behavior	Information about effects of the sun and sun protection recommendations: Background and recommendations on the prevention of skin cancer in children	Tailoring: information designed to meet staff’s stated needs of sun protection measures Facilitation: pointing at barriers to adequate sun protection and advice on overcoming these barriers Persuasive communication: Appeal to staff’s vocational goal of caring for children’s health and well-being Framing: emphasizing risks of inappropriate sun protection Consciousness raising: providing scientific background information about consequences of UV exposure Active learning: presentation of information is combined with opportunities for staff members to discuss experiences and habits
	Group discussion on consequences of excessive UV exposure and tanning attitudes	Discussion: Staff members are encouraged to discuss their attitudes and behavior in an open debate Self-reevaluation: encouraging reflection on knowledge and attitudes
	Joint planning of suitable sun- protection measures on behavioral and environmental level	Participation: joint discussion assures high level of engagement of staff members, which helps to promote changes in attitudes and behavior (individual level) Goal setting/implementation intentions: discussing and fixing goals and concrete behaviors for sun protection
Environmental changes in preschool	Development of an individual sun protection strategy	Participatory problem solving: staff team identifies current sun protection measures and develops a strategy for future measures Structural redesign: staff team reflects on organizational and technical elements that impede sun protection and finds ways to change them Public commitment: sun protection strategy is displayed in preschool, visible for staff and parents
Providing material for educational measures	Presentation and distribution of the CLEVER project week material	Facilitation: easy-to-use materials reduce barriers to sensitize children for sun protection

**Table 4:** Baseline demographic characteristics and baseline outcome scores of preschool staff.

	Intervention (*n* = 146)	Control (*n* = 127)
Demographic characteristics		
Age, mean (SD)	42.5 (12.43)	43.7 (12.52)
Gender, N females (%)	140 (96.6)	123 (96.9)
Education, N (%)		
Less than 10th grade	1 (0.7)	0 (0.0)
10th grade	85 (61.6)	76 (60.8)
Higher than 10th grade	52 (30.2)	49 (39.2)
Baseline outcome scores		
Behavioral outcomes, mean (SD)		
Sunscreen use	2.40 (0.81)	2.21 (0.81)
Sun avoidance	2.80 (0.51)	2.82 (0.43)
Psychosocial outcomes, mean (SD)		
Sunscreen use self-efficacy	3.11 (0.62)	3.06 (0.73)
Sun avoidance self-efficacy	3.03 (0.61)	3.11 (0.61)
Health-related outcome expectancies	3.40 (1.00)	3.26 (1.08)
Appearance-related outcome expectancies	2.50 (0.90)	2.53 (1.03)
Impediments to sunscreen use	0.73 (0.94)	0.97 (1.23)
Impediments to sun avoidance	1.65 (0.92)	1.41 (1.01)
Necessity of sun protection	3.08 (1.07)	3.07 (0.62)
Sun protection policy, mean (SD)	1.50 (1.18)	1.73 (1.23)

**Table 5:** Changes in behavioral and psychosocial outcomes and Sun Protection Policy scores after 1 month and 1 year.

Scale		Within Group Difference Intervention Group		Within Group Difference Control Group		Between Group Difference in Changes		
	Score Range	Baseline–1 Month Mean (95% CI) ^1^	Baseline–1 Year Mean (95% CI)^1^	Baseline–1 Month Mean (95% CI)^1^	Baseline–1 Year Mean (95% CI) ^1^	Mean (95% CI) ^1^	Test for Significance	Effect Size Hedges’ *d_B_* (95% CI)
Behavioral Outcomes								
Sunscreen Use	0–4		0.47 (0.25, 0.69)		0.46 (0.24, 0.68)	−0.01 (−0.40, 0.37)	*F*(1, 12.6) = 0.01, *p* = 0.95	−0.01 (−0.43, 0.40)
Sun-Avoidance	0–4		0.17 (0.05, 0.29)		−0.02 (−0.15, 0.10)	0.19 (0.02, 0.37)	*F*(1, 140) = 4.93, *p* ˂ 0.05	0.70 (0.04, 0.71)
Psychosocial Outcomes								
Sunscreen Use Self-Efficacy	0–4	0.40 (0.26, 0.54)		0.08 (−0.07, 0.23)		0.30 (0.02, 0.57)	*F*(1, 18.2) = 5.12, *p* ˂ 0.05	0.71 (0.03, 0.88)
Sun-Avoidance Self-Efficacy	0–4	0.29 (0.15, 0.43)		−0.20 (−0.35, −0.05)		0.49 (0.26, 0.72)	*F*(1, 9.8) = 22.38, *p* ˂ 0.001	1.09 (0.39, 1.07)
Health-Related Outcome Expectancies	0–4	0.24 (−0.03, 0.52)		0.39 (0.10, 0.68)		0.21 (−0.22, 0.65)	*F*(1, 15.7) = 0.40, *p* = 0.54	0.24 (−0.23, 0.69)
Appearance-Related Outcome Expectancies	0–4	0.01 (−0.21, 0.22)		0.00 (−0.22, 0.22)		0.00 (−0.35, 0.36)	*F*(1, 16.3) = 0.00, *p* = 0.99	0.00 (−0.29, 0.30)
Impediments to Sunscreen Use	0–4		−0.17 (−0.37, 0.03)		−0.15 (−0.36, 0.06)	−0.15 (−0.43, 0.13)	*F*(1,18.2) = 0.00, *p* = 0.97	−0.20 (−0.50, 0.15)
Impediments to Sun Avoidance	0–4		−0.32 (−0.52, −0.12)		0.11 (−0.09, 0.32)	−0.43 (−0.72, −0.15)	*F*(1,141) = 9.27, *p* < 0.01	−0.56 (−0.82, −0.17)
Necessity for Sun protection	0–4	0.36 (−0.07, 0.33)	-	0.23 (0.08, 0.37)	-	0.14 (−0.09, 0.36)	*F*(1,18.6) = 1.62, *p* = 0.22	0.35 (−0.14, 0.57)
Sun protection Policy	0–3		0.72 (0.38, 1.06)		0.59 (0.26, 0.91)	0.13 (−0.34, 0.60)	*F*(1,118) = 0.31, *p* = 0.58	0.08 (−0.26, 0.46)

^1^ Adjusted for baseline score and age.

**Table 6:** Baseline item ratings of preschool staff members (*n* = 273).

Scales and Items	Scale Mean (SD)	Item Median (Range)
Behavioral outcomes		
Sunscreen Use	2.31 (0.82)	
Put sunscreen on students when outside		3 (4)
Take sunscreen along on field trips		2 (4)
Use sunscreen for students UPF 30+		4 (4)
Reapply sunscreen every 2 h		2 (4)
Put sunscreen on myself when outside with students		4 (4)
Sun-Avoidance	2.81 (0.47)	
Students wear hats/caps when outside		2 (3)
Students wear tank tops/halter tops when outside^1^		2 (4)
Students wear long shorts/skirts when outside		2 (4)
Set up shaded areas outside and on field trips		4 (4)
Keep to shaded areas themselves and use protective clothing, when outside with students		3 (4)
Psychosocial outcomes		
Sunscreen Use Self-Efficacy	3.09 (0.67)	
Properly apply sunscreen		4 (4)
Ensure parents support provision/use of sunscreen		3 (4)
Take sunscreen on any occasion		3 (4)
Get more sunscreen when necessary		3 (4)
Sun-Avoidance Self-Efficacy	3.07 (0.61)	
Ensure parents provide protective clothing		3 (4)
Decide if area is sufficiently shaded		4 (4)
Check students wearing protective clothing		2 (4)
Provide spare clothes		4 (3)
Health-Related Outcome Expectancies	3.34 (1.04)	
Decreased risk for premature skin-aging		4 (4)
Decreased risk for skin cancer		4 (4)
Appearance-Related Outcome Expectancies	2.51 (0.96)	
Tanning makes me look better ^1^		2 (4)
Tanned skin is healthy skin ^1^		3 (4)
Impediments to Sunscreen Use	1.14 (0.84)	
It takes too much time		0 (4)
It is always messy		0 (4)
Impediments to Sun-Avoidance	1.29 (0.88)	
We do not have enough shade		0 (4)
Preventing taking of protective clothing is difficult		2 (4)
Necessity of Sun protection	3.08 (0.67)	
At the beach		4 (4)
At noon		4 (4)
On sunny evenings		2 (4)
On cloudy summer days		2 (4)
During outdoor sports activities		4 (4)
Sun Protection Policy	1.60 (1.20)	
Parents are requested to bring hats or protective clothing		1 (1)
Parents are requested to provide sunscreen or preschool supplies sunscreen		1 (1)
Parents are requested to provide permission for the use of sunscreen		0 (1)

^1^ Indicates item was reversed.

## Data Availability

The datasets used and/or analyzed during the current study are available from the corresponding author on reasonable request.

## Supplementary Materials

The following are available online at https://www.mdpi.com/article/10.3390/children8080651/s1, Figure S1: CLEVER-checklist for the development of the institutions’ individual sun protection strategy, Table S1: CONSORT 2010 checklist of information to include when reporting a randomised trial.
