# Supplementary material for: Cluster Randomized Trial: Sun Protection Intervention ‘Clever in Sun and Shade for Preschools’—Effectiveness and Dissemination
Source: Children (Basel). 2021 Jul 28;8(8):651. doi: 10.3390/children8080651 (PMC8391804; doi:10.3390/children8080651)
Supplement: Supplementary file 1 [file children-08-00651-s001.zip › children-1280907-supplementary.pdf]

Checkliste „Clever in Sonne und Schatten“

Unsere Sonnenschutz-Strategie

DER Sonnenschutz Clown

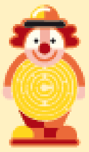

	Istzustand			Ziele		Umsetzung
	Das machen wir	Teilweise	Das machen wir nicht	Das wollen wir machen	Das wollen wir nicht machen	Das wollen wir so machen
**1. SONNEN-CHECK: Auf die Stärke der Sonne achten und Sonnenpausen einlegen**						
Sonnenschutz von April bis September: Sonnenschutz wird von Anfang April bis Ende September bedacht	✓	(✓)	✗	✓	✗	
Sonnenpause zwischen 11 Uhr und 15 Uhr: Von April bis September zwischen 11 und 15 Uhr im Haus oder im Schatten aufhalten	✓	(✓)	✗	✓	✗	
Schatten suchen: Beschattete Spielbereiche bevorzugen und bei wanderndem Schatten eventuell Spielbereich wechseln	✓	(✓)	✗	✓	✗	
Schatten in der Gartengestaltung einplanen: Bänke und Spielzeug an vorhandenen Schattenplätzen aufbauen, Schattenbäume oder Weidenrutenhütten pflanzen, Sonnensegel spannen, Tipis oder Buden mit den Kindern bauen	✓	(✓)	✗	✓	✗	
Schatten finanzieren: Bei Bedarf Finanzierungsmöglichkeiten schaffen: Haushaltsmittel, Spendenaufruf, Anfrage beim Träger, …	✓	(✓)	✗	✓	✗	
UV-Index: Zur Abschätzung der Stärke der UV-Strahlung informiert sich das Team in uneindeutigen Situationen, z. B. im April oder bei Bewölkung, über den aktuellen UV-Index (www.bfs.de/uv-prognose oder www.bfs.de/uv-aktuell)	✓	(✓)	✗	✓	✗	
**2. KLEIDUNGS-CHECK: Kleidung ist der beste Schutz**						
Schützende Kleidung: Kinder tragen luftige Oberteile, die mindestens die Schultern bedecken, und möglichst halblange Hosen oder Kleider	✓	(✓)	✗	✓	✗	
Kopfbedeckung: Kinder tragen Hüte oder Mützen mit einer breiten Krempe, die Gesicht, Ohren und Nacken schützen	✓	(✓)	✗	✓	✗	
Sonnenbrille: Kinder dürfen bei Bereitstellung durch Eltern im Freien Sonnenbrillen mit UV-Schutz tragen	✓	(✓)	✗	✓	✗	
Mitarbeit der Eltern: Eltern werden gebeten, T-Shirts, die die Schultern bedecken, und Kopfbedeckungen mitzugeben	✓	(✓)	✗	✓	✗	
Notfallkiste: Schützende T-Shirts und Hüte als Ersatz anschaffen, z. B. ‚entwachsene' Kleidungsstücke, kostenfreie ‚blaue Schirmmützen' der Deutschen Krebshilfe (Bestellung unter Tel.: 0228/72990-0 oder schaider@krebshilfe.de)	✓	(✓)	✗	✓	✗	
**3. SONNENCREME-CHECK: Alle unbedeckten Stellen eincremen**						
Geeignete Sonnenschutzcreme: Verwendung von Sonnencreme mit mindestens Lichtschutzfaktor 30, UVA- und UVB-Schutz sowie möglichst ohne Duft- und Konservierungsstoffe (bei Bedarf aktuelle Testergebnisse von Stiftung Warentest und Ökotest prüfen)	✓	(✓)	✗	✓	✗	
Ausreichend eincremen: Zwei gehäufte Teelöffel für das Gesicht, die Arme und den Nacken. Einen bis zwei gehäufte Esslöffel für den ganzen Körper eines Kindergartenkindes.	✓	(✓)	✗	✓	✗	
Nachcremen: Kinder früh und nachmittags eincremen sowie nach dem Baden oder Duschen	✓	(✓)	✗	✓	✗	
Sonnencreme bereitstellen: Jedes Kind bringt eine eigene Creme mit oder es gibt eine einheitliche Creme, welche durch Geldbeiträge der Eltern, Haushaltsmittel der Kita oder einen Förderverein finanziert wird; Ausnahmen bei Unverträglichkeiten oder Allergien ermöglichen	✓	(✓)	✗	✓	✗	
Elterneinverständnis: Bei Bedarf per Brief oder Aushang Einverständnis zum Eincremen der Kinder einholen	✓	(✓)	✗	✓	✗	
Unterstützung der Eltern: Eltern bitten, ihre Kinder am Morgen einzucremen und bei Bedarf ausreichend Sonnencreme mitzugeben	✓	(✓)	✗	✓	✗	
Notfallkiste: Vorrat an Sonnencreme anlegen für Kinder, die keine eigene Sonnencreme haben	✓	(✓)	✗	✓	✗	
**4. BILDUNGS-CHECK: Sonnenschutz zum Thema machen und mit gutem Beispiel vorangehen**						
Kinder: Sonnenschutz wird interaktiv behandelt (z. B. Bilder malen, Lieder singen, Schatten suchen)	✓	(✓)	✗	✓	✗	
Kita-Team: Erzieherinnen und Erzieher wissen um die Bedeutung des Sonnenschutzes und besprechen Maßnahmen bereits im April jeden Jahres	✓	(✓)	✗	✓	✗	
Eltern: Eltern werden über Elternbriefe oder einen Aushang über die Sonnenschutz-Strategie informiert und um Unterstützung gebeten	✓	(✓)	✗	✓	✗	
Vorbild sein: Erzieherinnen und Erzieher tragen z.B. schützende Hüte	✓	(✓)	✗	✓	✗	

CLEVER IN SONNE UND SCHATTEN

NCT

Deutsche Krebshilfe
HELFEN. FORSCHEN. INFORMIEREN.

DERMATOLOGISCHE PRÄVENTION

DIE SONNE UND WIR

Figure S1. CLEVER-checklist for the development of the institutions' individual sun protection strategy

Table S1 CONSORT 2010 checklist of information to include when reporting a randomised trial*

Section/Topic	Item No	Checklist item	Reported on page No
**Title and abstract**			
	1a	Identification as a randomised trial in the title	p. 1
	1b	Structured summary of trial design, methods, results, and conclusions (for specific guidance see CONSORT for abstracts)	p. 1
**Introduction**			
Background and objectives	2a	Scientific background and explanation of rationale	pp. 1-4
	2b	Specific objectives or hypotheses	p. 4
**Methods**			
Trial design	3a	Description of trial design (such as parallel, factorial) including allocation ratio	p. 4
	3b	Important changes to methods after trial commencement (such as eligibility criteria), with reasons	n.a.
Participants	4a	Eligibility criteria for participants	p. 4
	4b	Settings and locations where the data were collected	p. 4
Interventions	5	The interventions for each group with sufficient details to allow replication, including how and when they were actually administered	pp. 4-7
Outcomes	6a	Completely defined pre-specified primary and secondary outcome measures, including how and when they were assessed	pp. 7-8
	6b	Any changes to trial outcomes after the trial commenced, with reasons	n.a.
Sample size	7a	How sample size was determined	pp. 8-9
	7b	When applicable, explanation of any interim analyses and stopping guidelines	n.a.
Randomisation:			
Sequence generation	8a	Method used to generate the random allocation sequence	p. 9
	8b	Type of randomisation; details of any restriction (such as blocking and block size)	p. 9
Allocation concealment mechanism	9	Mechanism used to implement the random allocation sequence (such as sequentially numbered containers), describing any steps taken to conceal the sequence until interventions were assigned	p. 9
Implementation	10	Who generated the random allocation sequence, who enrolled participants, and who assigned participants to interventions	p. 9
Blinding	11a	If done, who was blinded after assignment to interventions (for example, participants, care providers, those assessing outcomes) and how	p. 9

	11b	If relevant, description of the similarity of interventions	n.a.
Statistical methods	12a	Statistical methods used to compare groups for primary and secondary outcomes	p. 9
	12b	Methods for additional analyses, such as subgroup analyses and adjusted analyses	p. 9
**Results**			
Participant flow (a diagram is strongly recommended)	13a	For each group, the numbers of participants who were randomly assigned, received intended treatment, and were analysed for the primary outcome	pp. 9-10
	13b	For each group, losses and exclusions after randomisation, together with reasons	pp. 9-10
Recruitment	14a	Dates defining the periods of recruitment and follow-up	p. 10
	14b	Why the trial ended or was stopped	n.a.
Baseline data	15	A table showing baseline demographic and clinical characteristics for each group	p. 11
Numbers analysed	16	For each group, number of participants (denominator) included in each analysis and whether the analysis was by original assigned groups	p. 12
Outcomes and estimation	17a	For each primary and secondary outcome, results for each group, and the estimated effect size and its precision (such as 95% confidence interval)	p. 12
	17b	For binary outcomes, presentation of both absolute and relative effect sizes is recommended	n.a.
Ancillary analyses	18	Results of any other analyses performed, including subgroup analyses and adjusted analyses, distinguishing pre-specified from exploratory	pp. 12-13
Harms	19	All important harms or unintended effects in each group (for specific guidance see CONSORT for harms)	p. 11
**Discussion**			
Limitations	20	Trial limitations, addressing sources of potential bias, imprecision, and, if relevant, multiplicity of analyses	p. 20
Generalisability	21	Generalisability (external validity, applicability) of the trial findings	pp. 17-19
Interpretation	22	Interpretation consistent with results, balancing benefits and harms, and considering other relevant evidence	pp. 17-19
**Other information**			
Registration	23	Registration number and name of trial registry	p. 1, p. 4
Protocol	24	Where the full trial protocol can be accessed, if available	n.a.
Funding	25	Sources of funding and other support (such as supply of drugs), role of funders	p. 19

*We strongly recommend reading this statement in conjunction with the CONSORT 2010 Explanation and Elaboration for important clarifications on all the items. If relevant, we also recommend reading CONSORT extensions for cluster randomised trials, non-inferiority and equivalence trials, non-pharmacological treatments, herbal interventions, and pragmatic trials. Additional extensions are forthcoming: for those and for up to date references relevant to this checklist, see www.consort-statement.org.
